# Exploring [^11^C]CPPC as a CSF1R-targeted PET imaging marker for early Parkinson’s disease severity

**DOI:** 10.1172/JCI186591

**Published:** 2025-04-15

**Authors:** Kelly A. Mills, Yong Du, Jennifer M. Coughlin, Catherine A. Foss, Andrew G. Horti, Katelyn R. Jenkins, Yana Skorobogatova, Ergi Spiro, Chelsie S. Motley, Robert F. Dannals, Wojciech G. Lesniak, Jae-Jin Song, Yu Ree Choi, Javier Redding-Ochoa, Juan C. Troncoso, Valina L. Dawson, Tae-In Kam, Martin G. Pomper, Ted M. Dawson

**Affiliations:** 1 Department of Neurology,; 2 Russell H. Morgan Department of Radiology and Radiologic Science,; 3 Department of Psychiatry and Behavioral Sciences,; 4 Neuroregeneration and Stem Cell Programs, Institute for Cell Engineering,; 5 Department of Pathology,; 6 Department of Physiology,; 7 Solomon H. Snyder Department of Neuroscience, and; 8 Department of Pharmacology and Molecular Sciences, Johns Hopkins University School of Medicine, Baltimore, Maryland, USA.

**Keywords:** Inflammation, Neuroscience, Innate immunity, Neurodegeneration, Parkinson disease

## Abstract

**BACKGROUND:**

Microglia-mediated brain immune changes play a role in the pathogenesis of Parkinson’s disease (PD), but imaging microglia in living people with PD has relied on positron emission tomography (PET) ligands that lack specificity in labeling immune cells in the nervous system. We aimed to develop imaging of colony stimulating factor 1 receptor (CSF1R) as a microglial-sensitive marker of innate immunity.

**METHODS:**

IHC using a CSF1R antibody evaluated colocalization with Iba-1 in PD (*n* = 4) and control (*n* = 4) human brain samples. Autoradiography using a CSF1R tritiated ligand in human brain samples from individuals with PD (*n* = 5) and in a control group (*n* = 4) was performed to obtain B_max_. PET imaging using a CSF1R radioligand was performed in 10 controls and 12 people with PD, and V_T_ was compared between groups and correlated with disease severity.

**RESULTS:**

IHC of CSF1R in human brain samples shows colocalization with Iba-1 and is significantly increased in brain samples from individuals with PD compared with individuals in a control group. Autoradiography revealed significantly increased CSF1R ligand binding in the inferior parietal cortex of patients with PD. [^11^C]CPPC PET showed higher binding in people with moderate PD compared with people in a control group and ligand binding correlated with more severe motor disability and poorer verbal fluency.

**CONCLUSION:**

This study underscores the significance of CSF1R imaging as a promising biomarker for brain immune function in Parkinson’s disease, which may be associated with cognitive and motor disease severity.

**FUNDING:**

PET imaging: the Michael J. Fox Foundation and the RMS Family Foundation. Radiotracer development: NIH (R01AG066464 and P41 EB024495). Postmortem brain tissues: NIH P30 AG066507 and BIOCARD study NIH U19 AG033655.

## Abstract 2

We evaluate the use of colony stimulating factor 1 receptor (CSF1R) as a marker of microglial-associated neuroinflammation in deceased and living people with Parkinson’s disease.

## Introduction

The involvement of the innate immune system, including microglial activation of neurotoxic astrocytes (1), is increasingly thought to play a pathogenic role in the cascade of events associated with α-synuclein (α-Syn) misfolding and neurodegeneration in Parkinson’s disease (PD). Signaling pathway abnormalities underlying microglial activation to disease-associated microglia converge with those found in genetic forms of PD (2) and idiopathic Parkinson’s disease (3, 4).

Based on clinicopathologic analyses of autopsy tissue (5) and the model of fibrillar α-Syn injection in mice (6), the spreading proteinopathy hypothesis holds that fibrillar α-Syn seeds and templates WT protein, causing toxic intracellular aggregation. Propagation of α-Syn fibrillation and cell-to-cell spread of pathologic α-Syn is driven, in part, by the innate immune response (7). Once oligomerized, fibrillar α-Syn activates microglia (8, 9), creating a cycle of immune cell dysfunction and neurodegeneration.

With the interplay between innate immunity and pathogenic α-Syn in the spread of pathologic α-Syn throughout the brain, suppressing this immune activation may allow disease modification in PD (7). A key step in developing targeted therapies to that end is generation of a specific, noninvasive biomarker for microglial activity and proliferation. To date, human in vivo assessment of CNS immune activation has primarily involved radiotracers targeting the translocator protein 18 kDa (TSPO) (10), but that approach has limitations. First generation TSPO ligands like [^11^C](*R*)-PK11195 have a poor signal-to-noise ratio and highly variable kinetic behavior (11). Newer TSPO radiotracers like [^11^C]DPA-713 have higher signal-to-noise ratios but are susceptible to low affinity in persons with a specific TSPO SNPs (12). More recently, it has also been shown that TSPO expression is not limited to microglia. For example, TSPO expression increases in astrocytes before microglia (13), may be present in neurons (14), and may increase with neuronal activation (15).

To sensitively measure microglial proliferation and activation with high cell type specificity, our team developed a radiotracer that targets colony stimulating factor 1 receptors (CSF1R), a tyrosine kinase involved in proliferation and activation of microglia, with high specificity in the inflamed state (16). While CSF1R may have a role in brain development through expression on specific neuronal populations (17) and microvascular cells (18), in the mature brain, the CSF1R is largely expressed by microglia (17) and is critical for their development and survival (19). The CSF1R radioligand, [^11^C]CPPC, has been shown to have faster kinetics and less off-target cerebellar binding than another leading candidate radiotracer, [^11^C]GW2580, which also shows radiotracer kinetics suboptimal for use in humans due to long scan times (20). [^11^C]CPPC regional volume of distribution (V_T_) values can be estimated in healthy individuals (21), suggesting its use as a radiotracer in immune-mediated disease states.

Given the need for in vivo assessment of microglial density and proliferation at early stages of neurodegeneration in PD to establish causality and potentially, as a biomarker for target engagement in disease modification trials, we studied [^11^C]CPPC in a cohort of persons with early PD and age-similar healthy participants in a control group. Our goal was to describe the relationship between PD disease severity and [^11^C]CPPC binding as a cell-type specific marker of microglia.

## Results

### Human postmortem studies

#### CSF1R IHC.

**Figure 1:**
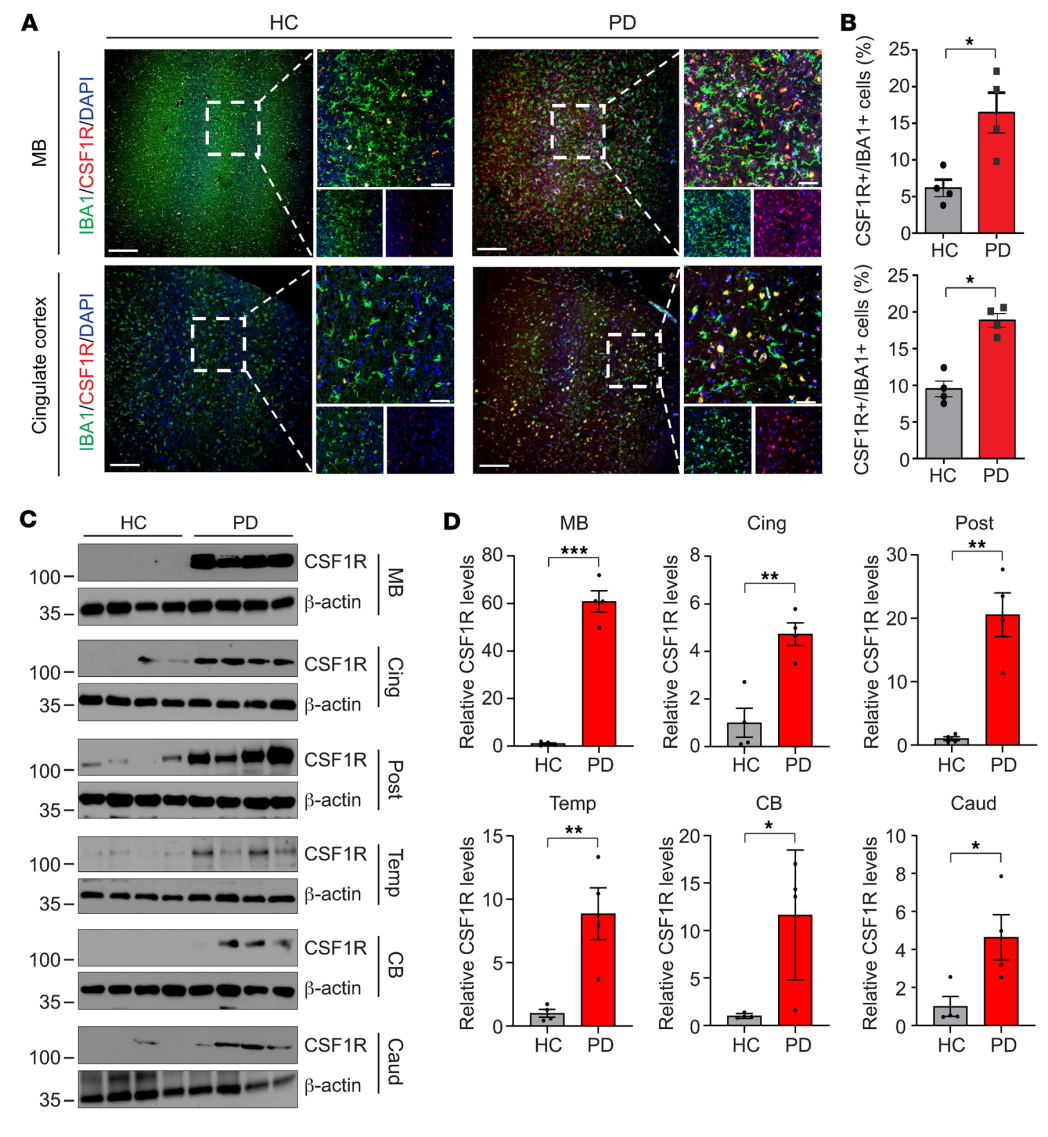
Increased levels of CSF1R in brains of human patients with PD (*N* = 4). (**A**) Representative confocal images with DAPI (blue), IBA1 (green), and CSF1R (red) in the midbrain and cingulate cortex of age-matched individuals who were in the healthy control and PD groups. White dashed lines indicate the region for high-magnification images. Scale bars: 200 μm (low-magnification images); 50 μm (high-magnification images). (**B**) CSF1R^+^ cells with IBA1^+^ cells are quantified (mean ± SEM; dots=individual data points); *P* values from independent, 2-tailed Student’s *t* tests. *P < 0.05, PD versus healthy control. (**C**) Representative immunoblots with CSF1R and β-actin antibodies in the midbrain (MB), cingulate cortex (Cing), posterior cingulate cortex (Post), temporal cortex (Temp), cerebellar cortex (CB) and caudate (Caud) of age-matched individuals who were in the healthy control and PD groups. (**D**) Relative CSF1R levels normalized to β-actin was quantified (*n* = 4). Individual data are shown as dots, bars are the mean ± SEM. *P* values were determined by unpaired 2-tailed Student’s *t* tests. *P < 0.05, **P < 0.005, ***P < 0.0005, PD versus healthy control.

IHC analysis revealed that CSF1R expression is significantly higher in a greater proportion of IBA1^+^ cells in persons with PD than in individuals in a control group (Figure 1, A and B). In addition, homogenized human brain tissue from people with PD showed consistently higher normalized CSF1R expression in the midbrain, cingulate cortex, posterior cingulate cortex, temporal cortex, cerebellar cortex, and caudate with the largest differences in the midbrain (*P* < 0.0005), compared with people in a controls group with absent neurodegenerative pathology (Figure 1, C and D). These results indicate that CSF1R is more highly expressed in established PD and that this expression colocalizes with another microglial marker.

#### B_max_ measurements.

**Figure 2:**
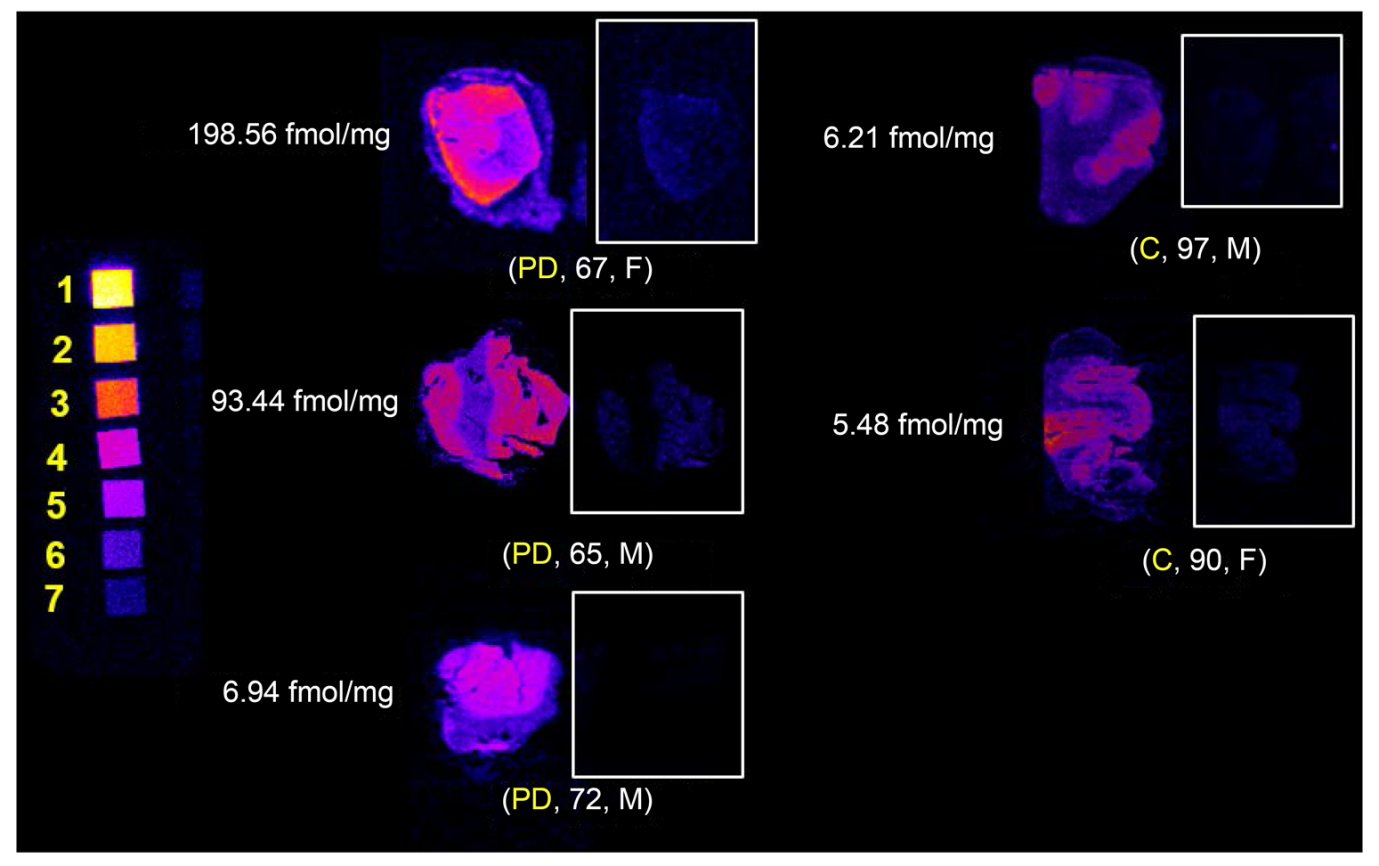
In vitro CSF1R autoradiography with [^3^H]JHU11761 in human inferior parietal cortex with and without Parkinson’s disease. Labels show diagnosis of Parkinson’s disease (PD) or control (C) with donor age and sex. Tritium scales standards on the left depict densities beginning at 5.89 nmol/g (1; yellow) and serially decrease until (7; blue), 0.09 nmol/g. Gray matter B_max_ is indicated to the left of each case.

**Figure 3:**
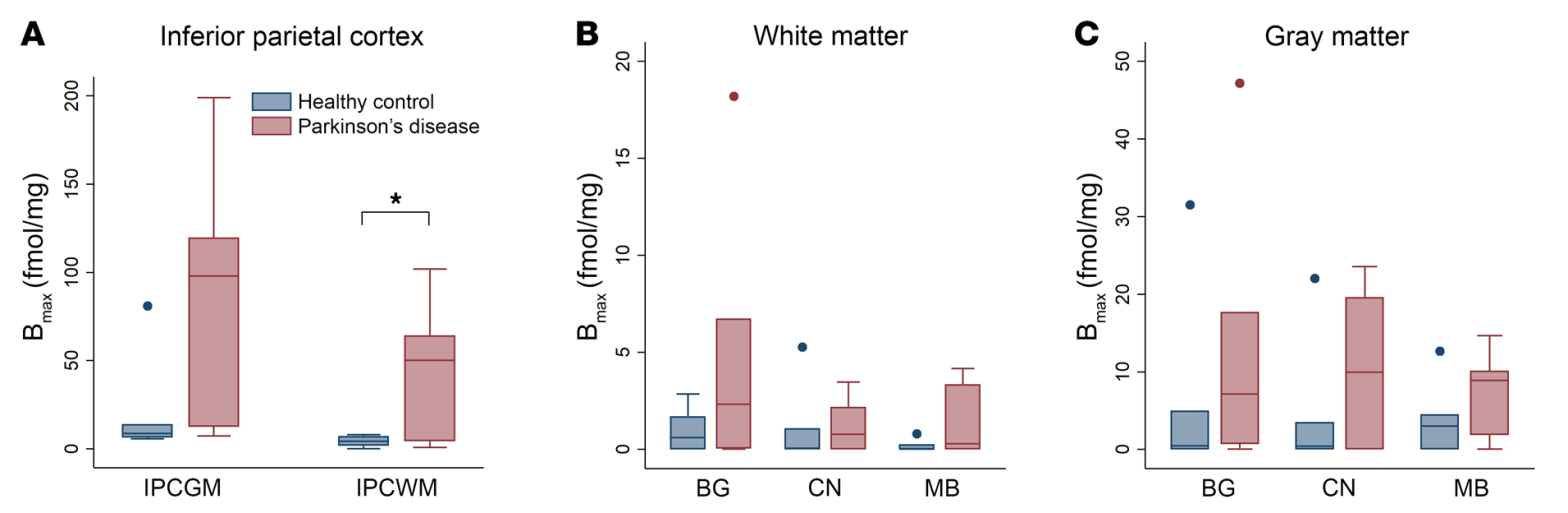
B_max_ of ^3^H-JHU11761 from human frozen sections in Healthy Controls (*N* = 6) or people with Parkinson’s disease (*N* = 6). Samples from (**A**) inferior parietal cortex gray matter (IPCGM) and white matter (IPCWM), and (**B**) white matter and (**C**) gray matter B_max_ in the caudate nucleus (CN), midbrain (MB), and basal ganglia (BG). Boxes indicate the interquartile (25th–75th) range, whiskers show 1.5 times interquartile range, the line in the box indicates the median and dots indicate outliers greater than or less than 1.5 times the interquartile range. IPCGM and IPCWM are shown separately due to difference in y-axis scale. Student’s independent *t* test was used for comparing B_max_ in HC vs PD, *P < 0.05.

In vitro autoradiography of PD and non-PD postmortem brain tissue was performed using the previously published tritiated CSF1R radiotracer 4-cyano-N-(4(4-[^3^H]methylpiperazin-1-yl)-2-(4-methylpiperidin-1-yl)phenyl)-1H-pyrrole-2-carboxamide ([^3^H]JHU11761) (22). Frozen sections were probed with serially diluted radiotracer with selected cases shown in Figure 2. B_max_ measurements were completed in sections of inferior parietal cortex (IPC), caudate nucleus (CN), midbrain (MB), and basal ganglia (BG), and are displayed in Figure 3 and Supplemental Table 1 (supplemental material available online with this article; https://doi.org/10.1172/JCI186591DS1). All values are corrected for a measured 3.65% free (79.35% serum protein bound) [^3^H]JHU11761 in the FBS used in these assays. Average gray matter uptake in PD IPC sections was 88.92 ± 6.83 fmol/mg compared with 20.56 ± 10.41 fmol/mg in control sections. Average white matter (WM) uptake in PD IPC was 45.10 ± 1.73 fmol/mg, while in control IPC, white matter uptake average is 5.01 ± 2.38 fmol/mg. PD uptake of probe in both regions was about 4–9 times higher than in control samples. No pattern was observed between male or female uptake. The average gray matter (GM) uptake in PD caudate nucleus was 15.72 fmol/mg while the average GM uptake in control caudate was 6.53 fmol/mg, a 241% increase in binding to PD caudate GM. WM uptake in PD caudate averaged 1.79 fmol/mg, while control WM uptake averaged 2.14 fmol/mg. Midbrain samples showed average PD GM uptake as 8.85 fmol/mg, while control GM binding averaged 5.73 fmol/mg, a 154% increase in the PD GM sections. WM uptake in PD midbrain averaged 2.68 fmol/mg, while control WM uptake in this region averaged 0.51 fmol/mg, nearly 5 times lower than PD WM uptake. PD GM uptake in basal ganglia averaged 15.95 fmol/mg, while control GM uptake averaged 12.41 fmol/mg, a nearly equal uptake in PD. In WM, PD uptake averaged 5.92 fmol/mg, while control WM uptake averaged 1.42 fmol/mg in basal ganglia. There was a statistically significant difference in [^3^H]JHU11761 uptake between participants in the control group and those with PD in the WM IPC (ρ = 0.047).

#### Equilibrium binding survey.

Supplemental Figure 1 shows the results of a 5 nM binding survey to elicit the number of CSF1R sites in inferior parietal cortex, midbrain, and caudate nucleus across four controls and 5 samples with PD. Three controls out of four showed low tracer binding in caudate putamen and midbrain and one PD case also showed low tracer binding across the three subregions. BRC2020 (control) had elevated binding in IPC and caudate nucleus. This may be a consequence of observed cerebrovascular disease and its associated inflammation. Four out of 5 PD cases showed elevated radiotracer binding across gray matter subregions, suggesting elevated CSF1R-related inflammation.

### CSF1R PET Imaging

We initially obtained consent from 12 individuals as part of a control group but excluded one due to recent COVID19 infection within the past 2 weeks and excluded another because of cognitive complaints within domains of memory and executive function. We obtained consent and enrolled 12 participants with PD, all with a Hoehn and Yahr score of 2 or lower and within 2 years of diagnosis. Of the 12 participants with PD, 5 had PD-MCI based on our criteria. In addition, of the 12 participants with PD, 8 had “mild” and 4 had “moderate” disability from motor symptoms based on whether they were equal to or below versus above the median MDS-UPDRS-II score, respectively. Clinical characteristics of these groups are in Table 1.

**Figure 4:**
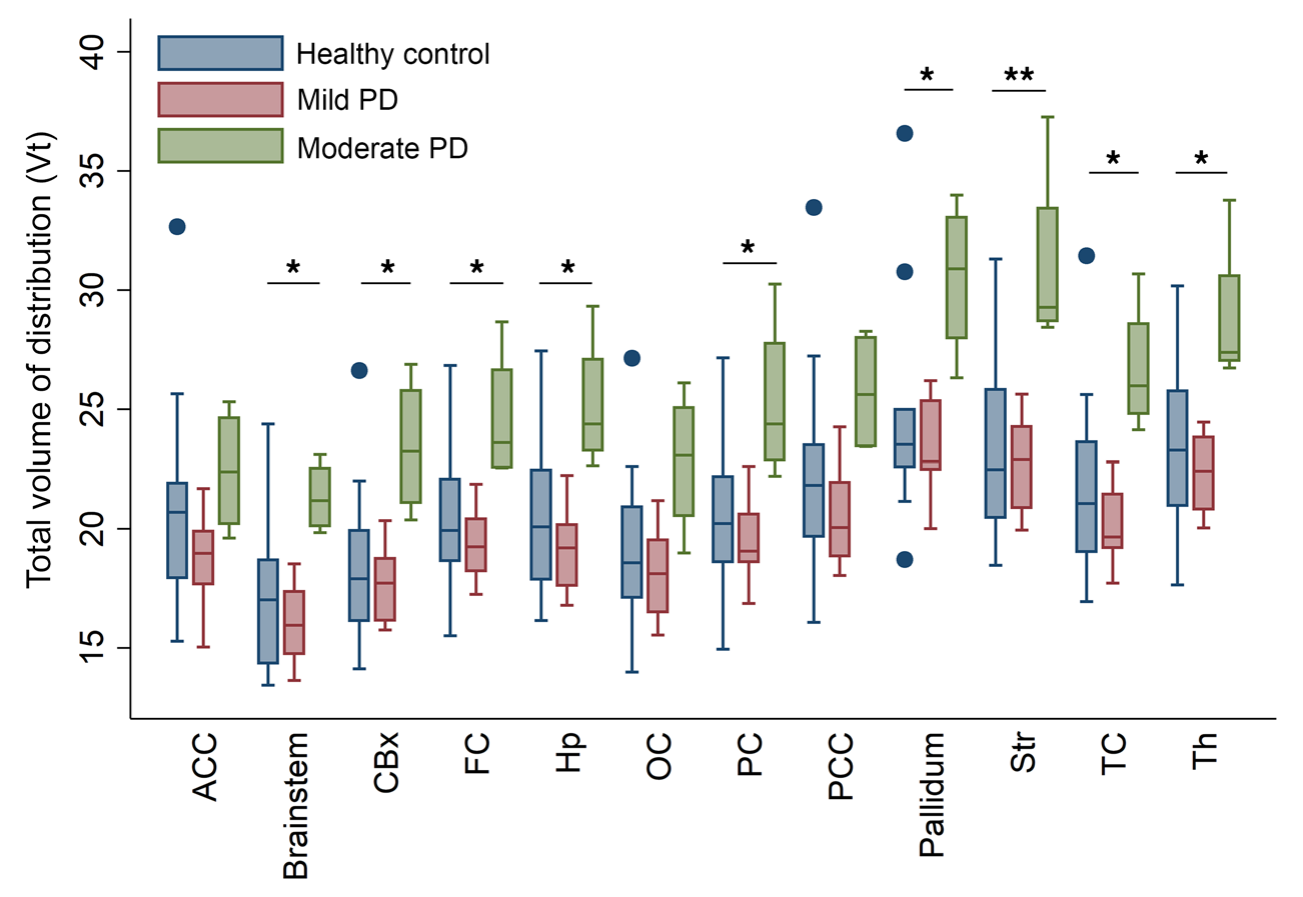
Regional total volume of distribution (V_T_) of [^11^C]CPPC, a CSF1R PET radioligand, in people with mild or moderate PD, defined by motor disability, and controls of a similar age. Boxes indicate the interquartile (25th–75th) range, whiskers show 1.5 times interquartile range, the line in the box indicates the median and dots indicate outliers greater than or less than 1.5 times the interquartile range. *P < 0.05 and **P < 0.004 for ANOVA and post hoc test between moderate PD (*N* = 4) and mild PD (*N* = 10) and or moderate PD vs healthy controls (*N* = 10). ACC, anterior cingulate cortex; CBx, cerebellar corte;, FC, frontal cortex; Hp, hippocampus; OC, occipital cortex; PC, parietal cortex; PCC, posterior cingulate cortex; Str, striatum; TC, temporal cortex; Th, thalamus.

**Figure 5:**
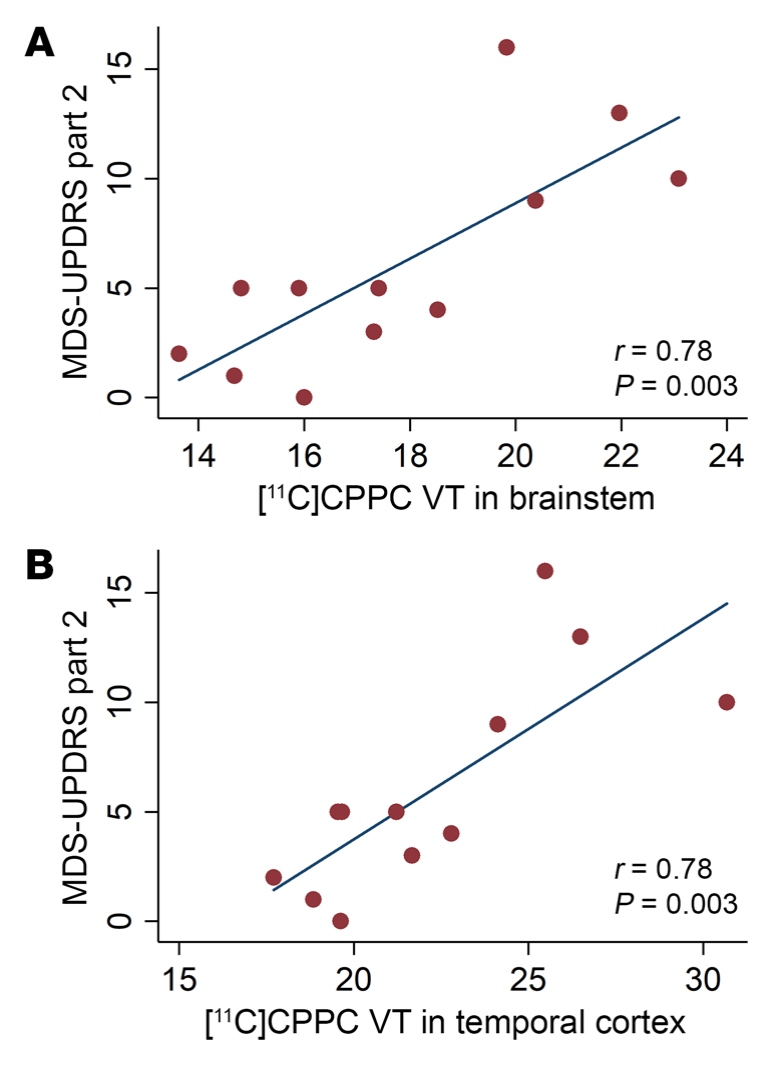
Correlation between regional [^11^C]CPPC V_T_ and motor disability in Parkinson’s disease. Scatter plots and linear relationship between [^11^C]CPPC V_T_ and ADL disability from motor symptoms in people with PD (*n* = 12), measured by MDS-UPDRS Part II in regions of interest that showed a statistically significant relationship (*P* < 0.005) using Pearson correlation, including (**A**) Brainstem and (**B**) Temporal cortex.

The mean [^11^C]CPPC V_T_ was computed using the Logan method and plotted across ROIs in individuals in a healthy control group and those with both mild- or moderate-PD based on disability from motor symptoms quantified by the MDS-UPDRS part II (Figure 4). ANOVA testing for the difference in mean V_T_ between these groups showed significant difference by group in the brainstem, cerebellar cortex, striatum, frontal cortex, hippocampus, pallidum, thalamus, and frontal, parietal, and temporal cortices, though the difference between groups was only statistically significant in the striatum when adjusting for multiple comparisons (*P* < 0.004) (Supplemental Table 2). Tukey’s post hoc testing showed that this difference was driven by the differences between the moderate motor disability and mild motor disability groups in the brainstem, cerebellar cortex, striatum, pallidum, hippocampus, thalamus, and frontal, parietal, and temporal cortices and between moderate motor disability and control groups in the cerebellar cortex, striatum, hippocampus, thalamus, and frontal and parietal cortices. No regions showed a statistically significant difference between people who were in the healthy control and mild motor disability groups in a group-wise comparison, but V_T_ in multiple regions showed a positive correlation with motor-related disability. Greater motor disability was associated with higher [^11^C]CPPC binding in the brainstem, cerebellar cortex, thalamus, hippocampus, striatum, pallidum, and temporal, occipital, frontal, posterior cingulate, and parietal cortices, though only the brainstem (*r* = 0.78, *P* = 0.003) and temporal cortex (*r* = 0.78, *P* = 0.003) remained statistically significant after adjusting for multiple comparisons (Table 2). Representative scatter plots from the brainstem and temporal cortex, fit with a linear representation of this correlation, are seen in Figure 5.

Regional [^11^C]CPPC VT was also assessed for correlation with the MDS-UPDRS Part III, the clinician-rated motor score. We did not find any statistically significant correlations, especially with adjusting α for multiple comparisons. There was a trend toward correlation between [^11^C]CPPC and MDS-UPDRS Part III in the striatum (*r* = 0.42, *P* = 0.175) and the pallidum (*r* = 0.54, *P* = 0.068). We attribute this discrepancy between the findings with Parts II and III of the MDS-UPDRS to the sensitivity of each subscale in the detection of disease severity in people with early PD, as has been shown in the PPMI cohort (23) and other prospective studies (24). The regions that showed a weak, nonstatistically significant correlation with Part III (striatum, pallidum) are regions most likely to be involved in early PD pathophysiology, suggesting biological plausibility of an association not detected in this specific cohort because of scale characteristics in this type of PD cohort.

Participants with PD were also stratified by cognitive status, and at a group level, [^11^C]CPPC was not higher in any region in participants with PD-MCI than those with PD-NC or those who were healthy controls (ANOVA *P* > 0.05 for all regions). Pearson correlation between regional [^11^C]CPPC V_T_ and a measure of global cognitive function, MoCA, were of moderate strength at best (putamen; *r* = –0.42, *P* = 0.18). Because this cohort has early PD, there was a ceiling effect of the MoCA with 4 of 5 patients with PD-NC having a 28 or 29 out of 30 total possible points. However, worse phonemic verbal fluency was associated with higher [^11^C]CPPC V_T_ in multiple regions of interest (Table 3), but statistical testing of these correlations did not survive correction for multiple comparisons with α = 0.004.

## Discussion

In response to the substantial heterogeneity in clinical presentation and pathogenic mechanisms in PD (25), precision medicine strategies are being sought to identify mechanistic subtypes of PD and target those subtypes with relevant disease-modifying strategies. Especially in sporadic PD, an increasing focus has been placed on the role of innate CNS immunity (26), with evidence of elevated innate immune activity in at least a subset of patients with PD (27). This study shows that, in human brain, CSF1R colocalizes with microglial markers and is elevated in autopsy tissue from patients with PD compared with individuals in a control group and presents data on the use of [^11^C]CPPC to measure CSF1R binding in early stage PD patients. This study adds to the growing literature on timing of microglial response in neurodegenerative disease by showing correlations between microglial proliferation and motor and cognitive disease severity in PD even at an early stage and that this can be measured with a novel radioligand targeting CSF1R, which has advantages over previously used PET ligands to detect inflammation in PD.

Both [^11^C]CPPC and [^3^H]JHU11761 (4-cyano-N-(4(4-[^3^H]methylpiperazin-1-yl)-2-(4-methylpiperidin-1-yl)phenyl)-1H-pyrrole-2-carboxamide) bind to CSF1R with high affinity (8.48 nM and 0.59 nM, respectively) and [^3^H]JHU11761 is structurally similar to CPPC. The unlabeled version of [^3^H]JHU11761 was described elsewhere (see compound 8 in Illig et al.) (28). Postmortem samples were probed with serially diluted 427 pM–427 fM binding stocks, well below CSF1R Ki values for both CPPC and JHU11761. Functionally, JHU11761 also inhibits several other kinases including (Kit, Axl, TrkA, FLT3, and IRK-β) (28), but with 5 times lower potency (IC_50_ = 3.5–83 nM) than for CSF1R (Ki = 0.59 nM). At high concentration (10 μM), unlabeled CPPC inhibits the same targets (29), making high specific activity a priority in PET studies with [^11^C]CPPC. Our autoradiography findings indicate that even in a small group of post mortem PD samples with low Alzheimer’s disease copathology, there was higher binding in the inferior parietal cortex compared with samples from age-similar controls. Notably, the one PD sample that showed low CSF1R binding had Lewy pathology that was restricted to the brainstem, whereas most other PD samples had limbic ± neocortical Lewy pathology (Figure 3 and Supplemental Table 1).

The human PET data are complementary to previous work that showed elevation of TSPO in PD participants versus those without known neurodegeneration using PET. By imaging mitochondrial activation within microglia, TSPO imaging offers different information on microglial states in vivo compared with CSF1R imaging. Several issues complicate the use of TSPO ligands in research and as potential biomarkers of innate immunity; though most highly expressed in activated microglia, TSPO may be increased in expression by activated astrocytes even before increased TSPO expression by microglia in neurodegeneration (13). TSPO may also be found in neurons where it increases with neuronal activation (15). While initial studies with [^11^C]-PK11195 showed higher binding in individuals in the PD versus control groups (30), a subsequent study showed overlap between individuals with PD and those who were healthy controls and that treatment with an antiinflammatory unexpectedly increased binding potential in the same brain regions (31). While not susceptible to genetic variation in TSPO binding affinity like later generation TSPO ligands, [^11^C]-PK11195 also has a low signal-to-noise ratio due to off-target binding (32). Later generation TSPO ligands like [^18^F]DPA-714 have higher affinity, but this affinity can be substantially reduced by a SNP in the TSPO gene. In a large study comparing [^18^F]DPA-714 binding in people with PD and those in the control group, even after persons with low affinity were excluded based on genotyping, significant differences in binding between people with PD and those in the control group were only found in the 40 high-affinity binders, but not in the 27 mixed-affinity binders (27). This indicates that use of new generation TSPO ligands to diagnose those with elevated innate immunity or to track microglial activation after an intervention would be limited to high-affinity binders, which are expected to comprise only 49% of the population (12). On the other hand, TSPO imaging has been performed in a number of autoimmune, infectious, and neurodegenerative diseases, allowing for comparison of relative immune activation across conditions. In our participants who were older, healthy controls, and in the least-affected PD group, the variance in binding was relatively small without outliers to suggest high natural variance in binding. Thus, our finding of a potentially more specific marker of microglial activation that is not known to have genetic variation in binding affinity for its target, represents an advance with a more specific marker of the microglial proliferation in PD. Future studies are needed to directly compare the sensitivity of [^11^C]CPPC and TSPO ligands to microglial activation in vivo.

While this study cannot establish a causal link between microglial activation and α-synuclein–related neurodegeneration, it aligns with preclinical and neuropathological evidence that supports a contributory role in cell death. Microgliosis precedes cell death in α-synuclein–overexpression animal models (33) and in human autopsy studies (26), and at least 2 mechanisms have been proposed: (a) direct or indirect (via activation of neurotoxic astrocytes) toxicity to neurons by α-synuclein–activated microglia (1, 26, 34) and (b) microglia-mediated enhancement of α-synuclein oligomerization (35) and spread throughout the brain (36, 37). Since some of the most promising disease-modifying agents in PD (38) are thought to act via microglia (7); having an in vivo biomarker for microglial activity may facilitate therapy development and hasten drug trials as an indicator of target engagement in proof-of-principle studies and clinical trials.

This study is not without limitations. Primarily, the relatively small sample size of 12 persons with PD in the PET experiment may limit generalizability of the findings. However, we would posit that the signal found in these data showing association with disease severity is useful in combination with the autopsy and autoradiography data to suggest more research focused on CSF1R ligands, such as [^11^C]CPPC, in understanding the role of microglia in neurodegeneration. Further, the sample was even smaller when stratified by sex, and this interaction between sex and CSF1R binding needs to be studied in larger cohorts of people with and without neurodegenerative conditions. Similarly, the finding of a difference in regional [^11^C]CPPC between participants with moderately severe PD relative to people in the control group, but not between people with mildly severe PD and people in the control group, could be due to high heterogeneity between groups in a small sample or could indicate a relative lack of microglial activation at the earliest stages of PD; the time course of microglial activation in PD will need to be explored in longitudinal studies. Also, we cannot rule out the possibility of copathology with amyloid β or τ in the patients with PD-MCI and plan to include these assessments in future studies. Other potential limitations have to do with decisions surrounding PET analysis. In this case, we chose not to apply correction for partial volume effects because the participants with PD had relatively mild disease where atrophy is not substantial and because of a lack of clarity around which method is best and whether PVC should be applied at all (39, 40).

In conclusion, these data suggest that using [^11^C]CPPC as a radioligand of CSF1R can detect microgliosis in early PD with at least moderate severity in patients, and that variance in [^11^C]CPPC binding within patients with PD correlates with disability from motor dysfunction and cognitive impairment. [^11^C]CPPC could be useful for identifying potential participants with microgliosis for trials that target immune activation in PD or for evaluating target engagements in such studies.

## Methods

### Sex as a biological variable.

Our study included autopsy samples and PET data from humans with PD. Given the size of the cohort used in this study to determine whether early signs of disease severity were associated with regional CSF1R binding, we did not consider sex as a biological variable due to the small sample size of females (*N* = 2); therefore, we were unable to stratify the analysis by sex. Similarly, the autopsy study sample size did not allow stratification by sex. Future studies with a larger sample will analyze the interaction between sex, CSF1R binding, and PD.

### Human postmortem brain tissues.

This research uses anonymous autopsy material; the frozen tissues and formalin-fixed sections of each brain regions from individuals with PD (*n* = 5) and age-matched people who were controls (*n* = 4) were obtained from the Brain Resource Center, Division of Neuropathology, Department of Pathology of Johns Hopkins University School of Medicine. Case diagnoses were adjudicated by consensus of movement disorders neurologists and neuropathologists at a multidisciplinary clinicopathological conference, and Lewy pathology was staged according to the McKeith criteria (41) (Supplemental Table 1). Alzheimer’s disease neuropathological change (ADNC) was described using the ABC scoring system (42).

### IHC.

IHC was performed on human brain sections including cingulate cortex and midbrain. Primary antibodies and working dilutions used were as follows: CSF1R (NBP2-37292, 1:100) and IBA1 (Wako 019-19741, 1:500). Brain sections were deparaffinized with 4 changes of xylene for 2 minutes each and hydrate in 4 changes of 100% ethanol for 2 minutes each and 4 changes of 95% ethanol for 1 minute each. After washing for 5 minutes, sections were incubated in peroxidase blocking solution, followed by being blocked with 4% donkey serum (Sigma-Aldrich) in PBS plus 0.2% Triton X-100 and incubated with primary antibodies (Supplemental Table 3). After brief washes with PBS containing 0.2% Triton X-100, brain sections were incubated with corresponding secondary antibodies conjugated with fluorescent dyes (Alexa Fluor 488–conjugated donkey antibody to rabbit IgG for IBA1; Alexa Fluor 568–conjugated donkey antibody to mouse IgG for CSF1R). The images were acquired by confocal scanning microscopy (LSM710, Carl Zeiss), followed by processed by the Zen software (Carl Zeiss). The total number of IBA1^+^ cells was determined by counting cells in three randomly selected regions per section. Colocalization was assessed by identifying cells with overlapping signals of IBA1 and CSF1R. All quantifications were performed in a blinded manner.

### Western blot analysis.

Human postmortem brain tissues were homogenized and prepared in lysis buffer [50 mM Tris-HCl (pH 7.4), 150 mM NaCl, 1 mM EDTA, 1% Triton x-100, 0.5% SDS, 0.5% sodium-deoxycholate, phosphatase inhibitor mixture I and II (Sigma-Aldrich), and complete protease inhibitor mixture (Roche)]. The homogenates were rotated at 4°C for 30 minutes for complete lysis, and centrifuged at 15,000*g* for 20 minutes. The supernatants were used for protein quantification using the BCA assay (Pierce). Samples were separated using SDS-polyacrylamide gels and transferred onto nitrocellulose membranes. The membranes were blocked with 5% non-fat milk in TBS-T (Tris buffered saline with 0.1% Tween-20) for 1 hour, probed using CSF1R antibody (Invitrogen, 14-1152-82), followed by incubation with the HRP-conjugated secondary antibodies (Cell Signaling) (Supplemental Table 3). The bands were visualized by ECL substrate.

### Autoradiography. B_max_ measurements.

Unfixed, frozen human brain tissues were obtained from the JHU Brain Resource Center from deaths occurring from PD or non-Parkinson’s/non-Alzheimer’s controls as has been previously published (22). Human autopsy tissues were cryosectioned to 16 μm thickness on charged glass slides and kept frozen at –80°C until use. Serially diluted solutions containing [^3^H]-JHU11761 (4-cyano-N-(4-(4-[3H]methylpiperazin-1-yl)-2-(4-methylpiperidin-1-yl)phenyl)-1H-pyrrole-2-carboxamide) in FBS (Sigma-Aldrich) spanned 9 nM through 9 pM. Each solution was applied to cognate slides and allowed to bind at ambient temperature for 10 minutes. Solutions were then rapidly aspirated off and the slides washed in ice cold PBS for 2 minutes. The PBS was aspirated off and any remaining moisture removed. The dry slides were then loaded into a Hypercassette (Amersham RPN-11647) and exposed to a charged phosphor screen (Cytiva, BAS-TR 2040) for 5 days. The screen was scanned using a Typhoon 9500IP Phosphor imager (Molecular Dynamics) and data visualized and quantitated using ImageJ software (Sourceforge.net). ROIs were drawn over gray and then white matter to quantitate radioligand uptake and then transformed with the tritium scales standard (ARC). Values were graphed using Prism software (GraphPad) to fit a Scatchard plot where the saturation binding value (B_max_) was obtained for each case and then as average PD and control values. Plasma protein binding of [3H]JHU11761 in lot-specific FBS was determined using 6 nM radioligand binding at ambient temperature followed by loading a 0.5 mL aliquot of FBS into an Amicon Ultra-0.5 mL 30K purification device, which was centrifuged at 14,000*g* for 30 minutes at room temperature. The filtrate was then saved, and the remaining supernatant was recovered by centrifugation at 1,000*g* for 2 minutes. The filtrate, recovered supernatant, and filter itself were primed with ScintiVerse scintillation cocktail and were counted separately in a Beckman Coulter LS 6500 multipurpose scintillation counter. The filtrate represented “free” or unbound radiotracer while the supernatant represented protein bound radiotracer (≥ 30 kDa protein bound). The filter device represented nonspecific binding and this value was subtracted from both supernatant and filtrate values. Percent free radiotracer was then used as a correction factor for measured B_max_ values. A 2-sided, 2 sample *t* test was applied to B_max_ averages between PD and control groups for each subregion as well as gray and white matter.

### Equilibrium binding survey.

Basal ganglia (containing caudate-putamen), midbrain and inferior parietal cortex containing slides (*n* = 3 and *n* = 1 blocking for each region and each case: 4 control, 5 PD) were obtained along with the B_max_ cohort and have the same properties. They were probed with 5 nM [^3^H]JHU11761 (22) in FBS or along with 50 μM unlabeled compound (blocking) in FBS for 10 minutes at ambient temperature. Longer ambient temperature binding invites loss of tissue integrity on the glass slides. Binding solution was aspirated and the tissues were washed with ice-cold PBS for 2 minutes. The PBS was then aspirated off and the slides were dried before loading into a Hypercassette for exposure to a charged phosphorscreen. Exposures took 5 days and were processed identically to the slides for B_max_ measurements, although quantitation involved only ROI drawing and comparison with the standard curve obtained from the tritium microscales.

### PET imaging participants.

We enrolled persons between ages 50–80 with a clinically established Parkinson’s disease (43) within 2 years of diagnosis and with Hoehn and Yahr stage 2 or lower. People in this state of disease are fully ambulatory and mostly independent. Healthy individuals, age 50–80, were recruited from PD and PD caregiver support groups, families of people with PD, or the general public. We excluded potential participants with other systemic autoimmune disorders, recent infection, antiinflammatory or immunosuppressant use, a history of moderate or severe TBI, or other known neurodegenerative diseases. Safety labs, including chemistry panel, liver function tests, serum pregnancy test (if female and premenopausal), and EKG were performed, and patients were excluded for lab values more than 1.5 times the upper limit of normal. The study was performed with approval by the Johns Hopkins Institutional Review Board and was compliant with the Declaration of Helsinki. Patients provided informed consent prior to participation in the study protocol.

At the baseline visit, all participants underwent examination for parkinsonism, and, if absent, the Prodromal PD Calculator was administered and individuals who were healthy controls were only enrolled as such if the likelihood ratio for prodromal PD did not meet or exceed that which would provide 80% likelihood of eventual PD, stratified by age (44, 45), and if they did not meet criteria for clinically established Parkinson’s disease (43). Participants with a prior diagnosis of PD were examined for parkinsonism and if present, the Movement Disorder Society Clinical Diagnostic Criteria for Parkinson’s Disease were applied. Those meeting criteria for clinically established Parkinson’s disease were enrolled in the Parkinson’s disease cohort.

### Clinical assessments.

All participants underwent the Movement Disorders Society-Unified Parkinson’s Disease Rating Scale (MDS-UPDRS) parts I–IV in the medicated state. To explore the relationship between disease severity and CSF1R imaging, participants were classified as “mild PD” if their MDS-UPDRS Part II was at the median or lower, or “moderate PD” if above the median. Part II was used because it is more sensitive to disease progression across the entire spectrum of disease severity, including early disease (24, 46), and is more predictive of motor progression to Hoehn and Yahr stage IV than the MDS-UPDRS Part III (23). As an exploratory analysis, ROI [^11^C]CPPC V_T_ was assessed for correlation with MDS-UPDRS Part III, though this was predicted to be less sensitive to differences in disease severity in this cohort with early PD. Stratification of this variable was justified by the large degree of variation (SD = 4.9 points) around the mean (6.2 points) and the observation that the measure of central tendency did not differ between cognitive groups and thus was assumed to be relatively independent (PD-NC mean MDS-UPDRS Part II = 6.2, PD-MCI mean = 6.2). Other assessments included Montreal Cognitive Assessment (MoCA), phonemic (letter) verbal fluency, Beck Depression Rating Scale-2, University of Pennsylvania Smell Identification Test, in addition to a medical history and medication history. Participants with PD were classified as having mild cognitive impairment (PD-MCI) by level 1 criteria (47) if they had a subjective report of cognitive impairment (MDS-UPDRS part 1, question 1 of at least 1) and an abnormal measure of global cognitive impairment (MoCA less than 26).

### MRI.

Participants received a brain MRI for PET coregistration and to screen for anatomic variations or lesions that would preclude analysis of PET data at a group level. MRI’s were performed on a Philips dStream Ingenia Elition 3T with a 3D-acquired T1-weighted sequence, T2 sequence, and iron-sensitive sequences. FreeSurfer (48) image analysis suite was used for automated segmentation of MRI into regions of interest (ROIs) in PD: brainstem, cerebellum, thalamus, hippocampus, temporal cortex, occipital cortex, frontal cortex, parietal cortex, pallidum, striatum, anterior cingulate cortex, and posterior cingulate cortex.

### PET imaging.

The synthesis of [^11^C]CPPC occurred in the Johns Hopkins PET Center, where radiochemical purity was confirmed at greater than 95% (16). The target injection radioactivity was 740 MBq, with a max of 777 MBq and minimum of 703 MBq. Participants arrived after an overnight washout of anti-parkinsonian medications and were fitted with a thermoplastic face mask to minimize head motion. A radial arterial catheter was inserted for serial arterial blood sampling and an intravenous catheter was inserted for PET radiotracer injection. A 128-slice low-dose CT was administered for attenuation correction before the injection. A 90-minute emission scan on a Siemens Biograph mCT PET/CT began with IV injection of 740 MBq of [^11^C]CPPC. Serial arterial blood was at first drawn as fast as possible for 90 seconds, then at increasingly longer intervals between blood samples thereafter. Select samples at 5, 10, 20, 30, 60, and 90 minutes were analyzed with high performance liquid chromatography (HPLC) for radioactive plasma metabolites (21). Thirty frames (4 of 15 seconds, 4 of 30 seconds, 3 of 1 minute, 2 of 2 minutes, 5 of 4 minutes, and 12 of 5 minutes) from the 90 minute emission scan were reconstructed using the iterative ordered subset expectations maximization algorithm and corrected for attenuation, randoms, scatter, and time-of-flight. PET data processing and tracer kinetic modeling were performed as previously described (21) using PMOD (v3.7, PMOD Technologies Ltd.). Briefly, interframe motion correction was first applied by frame-by-frame matching to a reference frame generated from the average of the frames from 30–60 minutes postinjection. Next, the PET data were transformed to MRI space through rigid registration. The MRI segmented ROIs were then applied to the registered PET images to generate a time activity curve for each region. The total distribution volume V_T_, which reflects the equilibrium ratio of [^11^C]CPPC concentration in tissue to the concentration in arterial plasma was finally calculated using Logan graphical analysis (49) with metabolite corrected plasma input function.

### Statistics.

For IHC, the mean percentage of cells in each sample with CSF1R^+^/IBA1^+^ staining was compared with unpaired 2-tailed Student’s *t* tests for each region. For immunoblotting results, CSF1R levels were reported as the mean CSF1R level normalized to β-actin and compared between PD and control groups using a 2-tailed Student’s *t* test. For human PET imaging, Pearson correlation was performed between age and clinical outcome variables including MDS-UPDRS Part II, MoCA, and verbal fluency, and, since these were not significant, future associations between clinical and imaging variables were unadjusted. Independent sample *t* tests were used to evaluate the difference in V_T_ between healthy controls and participants with PD in each ROI. Given a high degree of heterogeneity in regional V_T_ in the PD group, for each region of interest, [^11^C]CPPC V_T_ was correlated with disease severity measured by the MDS-UPDRS Part II, the most clinically relevant measure of disease severity in a prespecified posthoc analysis (46). Region of interest [^11^C]CPPC V_T_ was also assessed for correlation with MDS-UPDRS Part III. Based on findings from this analysis, PD participants were stratified in the in the domain of disability from motor symptoms into “mild” disease (less than or equal to the median MDS-UPDRS Part II score of 5) or “moderate” disease (greater than the median MDS-UPDRS Part II score of 5) in nonprespecified analysis. One-way ANOVA was performed for each region of interest to test for any difference in variance of V_T_ between 3 groups: healthy controls, “mild” PD, or “moderate” PD in a prespecified analysis. To further explore the heterogeneity of [^11^C]CPPC V_T_ in PD participants as it relates to variance in the cognitive domain, regional V_T_ was assessed for correlation with verbal fluency, a cognitive function known to involve multiple brain regions and that is affected early in PD (50).

Analyses were adjusted for multiple comparisons of 13 ROI’s using Bonferroni correction, with α set at 0.004. Given that this is a first-in-PD pilot study using [11C]CPPC PET to image the CSF1R, the statistical significance of unadjusted results were also presented. Analyses were performed with STATA (StataCorp. 2015. Stata Statistical Software: Release 14. College Station, Texas: StataCorp LP).

### Study approval.

The study protocol was approved by the Johns Hopkins University Institutional Review Board and conforms with the Declaration of Helsinki, as well as Good Clinical Practice guidelines. Participants participated after informed consent was documented in writing.

### Data availability.

The primary data used for PET analyses (regional V_T_), Western blot results, and values for all data points in figures are reported in the Supporting Data Values file.

## Author contributions

KAM, MGP, and TMD designed the study. JMC, CAF, AGH, WGL, JJS, YRC, JRO, TIK, YD, JCT, and RFD contributed to execution of the study procedures and manuscript drafting. ES, CSM, YS, and KRJ coordinated PET study visits and contributed to manuscript drafting. KAM executed the initial draft of the manuscript with executive editing by VLD and TMD.

## Supplementary Material

Supplemental data

ICMJE disclosure forms

Unedited blot and gel images

Supporting data values

## Figures and Tables

**Table 3:**
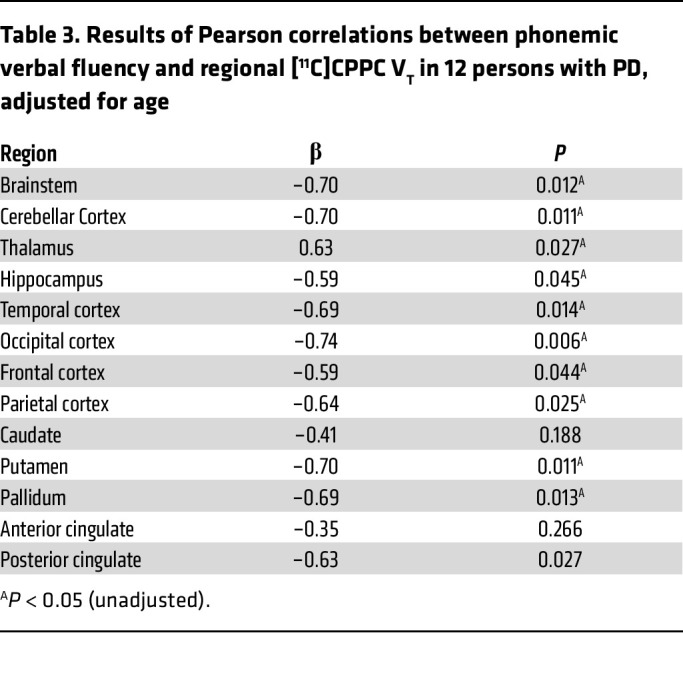
Results of Pearson correlations between phonemic verbal fluency and regional [^11^C]CPPC V_T_ in 12 persons with PD, adjusted for age

**Table 2:**
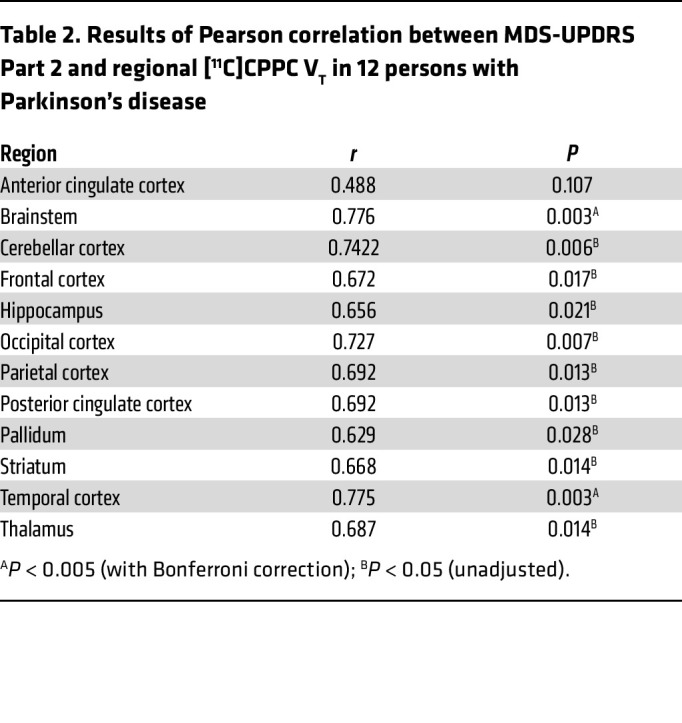
Results of Pearson correlation between MDS-UPDRS Part 2 and regional [^11^C]CPPC V_T_ in 12 persons with Parkinson’s disease

**Table 1:**
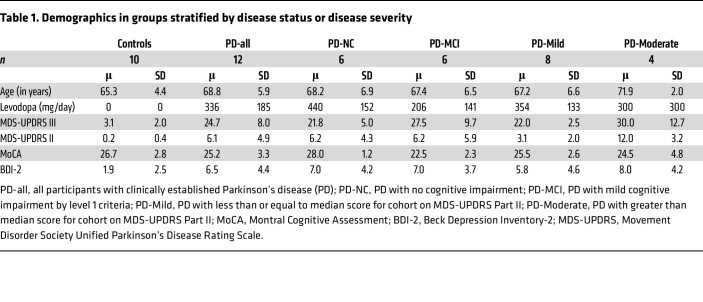
Demographics in groups stratified by disease status or disease severity

## Version 1 04/15/2025

In-Press Preview

## Version 2 06/16/2025

Electronic publication
