# Supplementary material for: Exploring [11C]CPPC as a CSF1R-targeted PET imaging marker for early Parkinson’s disease severity
Source: J Clin Invest. 2025 Apr 15;135(12):e186591. doi: 10.1172/JCI186591 (PMC12165784; doi:10.1172/JCI186591)
Supplement: Supplemental data [file jci-135-186591-s087.pdf]

Exploring [^{11}C]CPPC as a CSF1R-targeted PET Imaging Marker for Early Parkinson's Disease Severity.

Supplementary Data

Supplementary table 1.

Group	Sex	Age	PMI	Lewy Path	ADNC	Other	Inferior parietal cortex		Caudate		Midbrain		Basal ganglia	
							GM	WM	GM	WM	GM	WM	GM	WM
Control	F	90	22	None	None; A0B0C0		5.5	ND	0.7	ND	4.4	ND	0.9	0.5
Control	M	72	20	None	None; A0B0C0		8.8	8.0	ND	ND	ND	ND	ND	ND
Control	F	65	23	None	None; A0B0C0		80.7	4.2	ND	ND	ND	ND	ND	ND
Control	M	82	15	None	None; A0B0C0	CVD	13.6	6.7	22.0	5.3	12.6	ND	31.5	2.8
Control	F	68	12	None	None; A0B0C0		8.3	1.9	3.4	1.0	1.7	0.2	4.9	0.7
Control	M	97	16	None	None; A0B0C0		6.4	4.3	0.0	0.1	4.2	0.8	ND	1.66
PD	M	77	16	Neocortical	Low; A1B1C1		119.1	63.7	1.9	0.5	1.9	0.6	0.7	0.0
PD	M	91	19	Neocortical	Low; A1B2C1	CVD	12.6	4.4	18.0	2.1	14.6	4.2	17.6	6.7
PD	M	72	15	Brainstem	None; A0B0C0	Hypoxic-ischemic	7.3	0.7	ND	ND	8.1	ND	3.9	0.9
PD	M	65	6	Limbic	Very Low; A1B0C0	CVD + AA	94.4	44.3	19.5	1.0147	10.0	3.3	10.4	3.7
PD	F	67	16	Neocortical	Low; A1B1C2		198.8	101.9	ND	ND	ND	ND	ND	ND
PD	M	90	16	Brainstem	Low; A1B1C1	CVD	101.2	55.6	23.5	3.5	9.6	ND	47.1	18.2

B_{max} (fmol/mg) sites of ^{3}H-JHU11761 (CSF1R) in frozen sections. GM, grey matter; WM, white matter. ND=not detected. CVD = microvascular ischemic disease. AA = amyloid angiopathy. Lewy pathology = brainstem, limbic, or neocortical. ADNC = Alzheimer's disease related change (very low, low, moderate, high). PMI = Post Mortem Interval.

Supplementary table 2.

Region	F	p			t	p	
Anterior cingulate	1.65	0.217		HC vs. Mild PD	-2.56	0.338	
				HC vs. Mod PD	1.13	0.866	
				Mild PD vs. Mod PD	3.69	0.263	
Brainstem	5.25	0.015	*	HC vs. Mild PD	-1.4	0.522	
				HC vs. Mod PD	3.88	0.060	
				Mild PD vs. Mod PD	5.28	0.012	*
Cerebellar Cortex	5.59	0.012	*	HC vs. Mild PD	-1.02	0.738	
				HC vs. Mod PD	4.74	0.031	*
				Mild PD vs. Mod PD	5.77	0.011	*
Striatum	9.68	0.001	**	HC vs. Mild PD	-0.68	0.902	
				HC vs. Mod PD	7.68	0.002	**
				Mild PD vs. Mod PD	8.35	0.002	**
Frontal cortex	5.36	0.014	*	HC vs. Mild PD	-1.13	0.646	
				HC vs. Mod PD	4.11	0.043	*
				Mild PD vs. Mod PD	5.24	0.012	*
Hippocampus	6.39	0.008	*	HC vs. Mild PD	-1.47	0.519	
				HC vs. Mod PD	4.59	0.031	*
				Mild PD vs. Mod PD	6.05	0.006	*
Occipital cortex	3.24	0.062		HC vs. Mild PD	-1.22	0.677	
				HC vs. Mod PD	3.46	0.156	
				Mild PD vs. Mod PD	4.68	0.051	
Parietal cortex	5.75	0.011	*	HC vs. Mild PD	-0.99	0.748	
				HC vs. Mod PD	4.81	0.027	*
				Mild PD vs. Mod PD	5.80	0.010	*
Posterior cingulate	2.69	0.094		HC vs. Mild PD	-2.08	0.477	
				HC vs. Mod PD	3.15	0.341	
				Mild PD vs. Mod PD	5.23	0.079	
Pallidum	4.39	0.027	*	HC vs. Mild PD	1.48	0.716	
				HC vs. Mod PD	5.60	0.068	
				Mild PD vs. Mod PD	7.08	0.023	*
Temporal cortex	5.58	0.012	*	HC vs. Mild PD	-1.86	0.457	
				HC vs. Mod PD	4.69	0.057	
				Mild PD vs. Mod PD	6.55	0.009	*
Thalamus	6.48	0.007	*	HC vs. Mild PD	-1.13	0.713	
				HC vs. Mod PD	5.36	0.019	*
				Mild PD vs. Mod PD	6.49	0.006	*

F values for ANOVA comparing regional [^{11}C]CPPC V_T across healthy controls (HC), PD patients with mean MDS-UPDRS part II below median (Mild PD), and PD patients with mean MDS-UPDRS part II at median or

above (Moderate PD), all with Hoehn & Yahr stage ≤2. T values for pair-wise post hoc tests between groups with Tukey's test. *p<0.05 (uncorrected). **p<0.005 (corrected for multiple comparisons).

Supplementary table 3. Antibody identification for IHC and western blot experiments.

Target	Antibody	Source Company	Catalog Number
IHC			
CSF1R	M-CSFR/CD115 (6B9B9)	NOVUS Biologicals	NBP2-37292
IBA1	Anti-Iba1	Wako	019-19741
anti-Rabbit IgG	Goat anti-Rabbit IgG (H+L) Cross-Adsorbed Secondary Antibody	Alexa Fluor	488
anti-Mouse IgG	Goat anti-Mouse IgG (H+L) Cross-Adsorbed Secondary Antibody	Alexa Fluor	594
Western Blot			
CSF1R	CD115 (c-fms) Monoclonal Antibody	Invitrogen	14-1152-82
Actin	beta Actin Antibody (C4)	Santa Cruz	sc-47778
anti-Mouse IgG	Peroxidase AffiniPure® Goat Anti-Mouse IgG (H+L)	Jackson ImmunoResearch	115-035-146

Supplementary Figure 1.

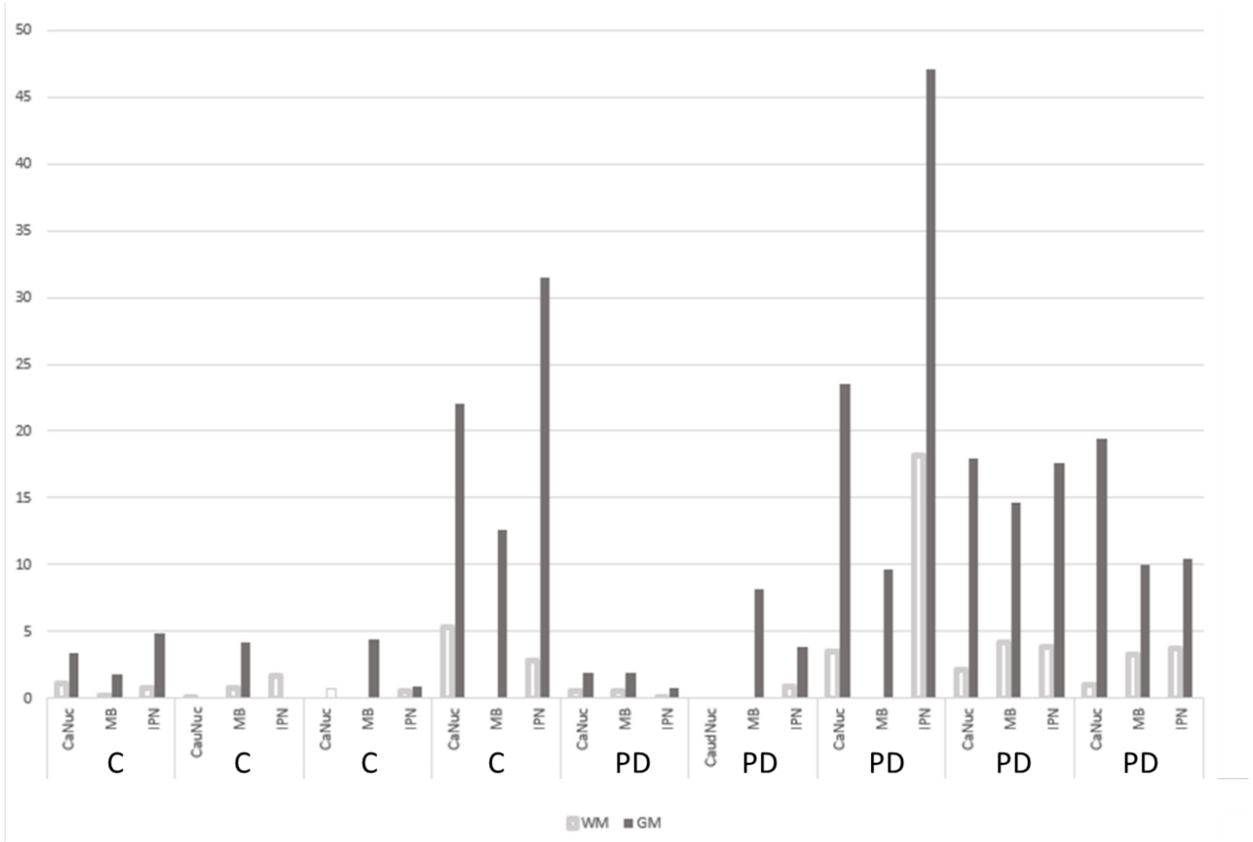

5 nM binding survey of ^{3}H-JHU11761 binding in 5 healthy controls (C) and 4 subjects with Parkinson's disease (PD) in inferior parietal cortex (IPC), midbrain (MB), and caudate nucleus (CaNuc).
