# Supplementary figures and images for: Exploring [11C]CPPC as a CSF1R-targeted PET imaging marker for early Parkinson’s disease severity
Source: J Clin Invest. 2025 Apr 15;135(12):e186591. doi: 10.1172/JCI186591 (PMC12165784; doi:10.1172/JCI186591)

Full unedited gels for Figure 1B, cropped rows marked with a box.

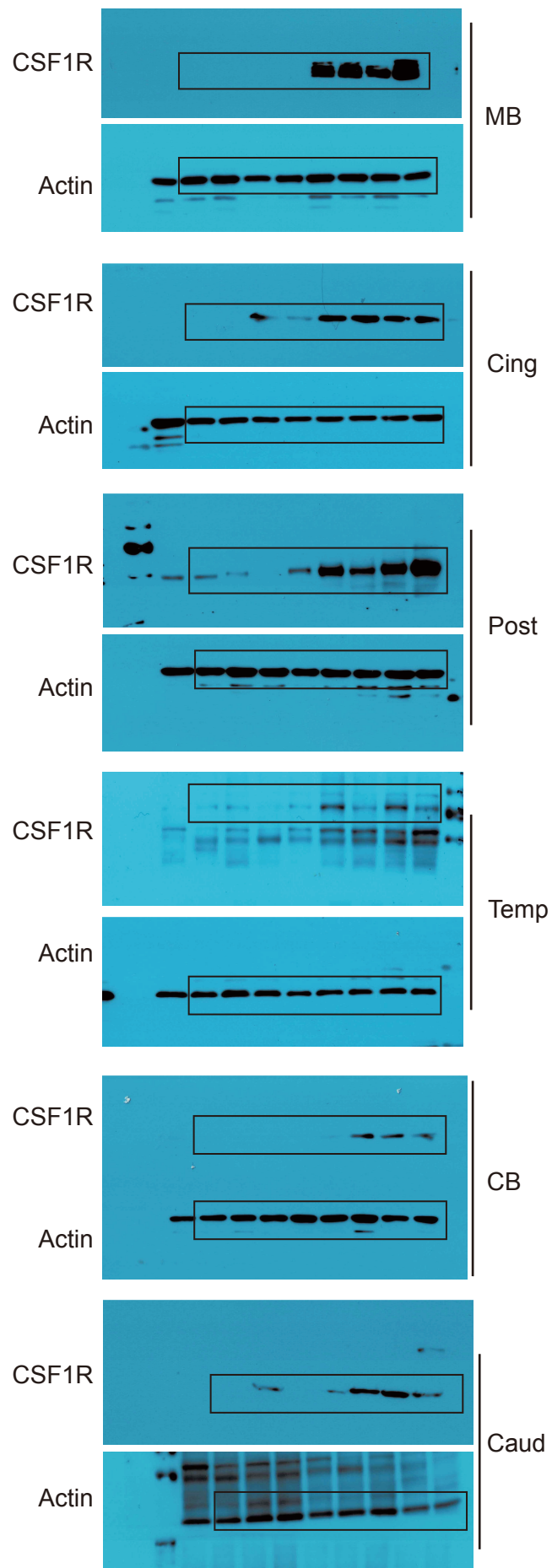

Supplement: Unedited blot and gel images [file jci-135-186591-s089.pdf]
